# Instrumented balance and walking assessments in persons with multiple sclerosis show strong test-retest reliability

**DOI:** 10.1186/s12984-017-0251-0

**Published:** 2017-05-22

**Authors:** Jordan J. Craig, Adam P. Bruetsch, Sharon G. Lynch, Fay B. Horak, Jessie M. Huisinga

**Affiliations:** 1 Landon Center on Aging, University of Kansas Medical Center, 3901 Rainbow Blvd., Mail Stop 1005, Kansas City, KS 66160 USA; 2 Bioengineering Graduate Program, University of Kansas, 3135A Learned Hall, 1530 W 15th St, Lawrence, KS 66045 USA; 3 Department of Neurology, University of Kansas Medical Center, 3901 Rainbow Blvd., Mail Stop 2002, Kansas City, KS 66160 USA; 4 Department of Neurology, Oregon Health & Science University, 3181 SW Sam Jackson Park Road, L226, Portland, Oregon 97239-3098 USA

**Keywords:** Balance, Walking, Accelerometers, Wireless sensors, Multiple sclerosis, Reliability

## Abstract

**Background:**

There is a need for objective movement assessment for clinical research trials aimed at improving gait and balance in persons with multiple sclerosis (PwMS). Wireless inertial sensors can accurately measure numerous walking and balance parameters but these measures require evaluation of reliability in PwMS. The current study determined the test-retest reliability of wireless inertial sensor measures obtained during an instrumented standing balance test and an instrumented Timed Up and Go test in PwMS.

**Methods:**

Fifteen PwMS and 15 healthy control subjects (HC) performed an instrumented standing balance and instrumented Timed Up and Go (TUG) test on two separate days. Ten instrumented standing balance measures and 18 instrumented TUG measures were computed from the wireless sensor data. Intraclass correlation coefficients (ICC) were calculated to determine test-retest reliability of all instrumented standing balance and instrumented TUG measures. Correlations were evaluated between the instrumented standing balance and instrumented TUG measures and self-reported walking and balance performance, fall history, and clinical disability.

**Results:**

For both groups, ICCs for instrumented standing balance measures were best for spatio-temporal measures, while frequency measures were less reliable. All instrumented TUG measures exhibited good to excellent (ICCs > 0.60) test-retest reliability in PwMS and in HC. There were no correlations between self-report walking and balance scores and instrumented TUG or instrumented standing balance metrics, but there were correlations between instrumented TUG and instrumented standing balance metrics and fall history and clinical disability status.

**Conclusions:**

Measures from the instrumented standing balance and instrumented TUG tests exhibit good to excellent reliability, demonstrating their potential as objective assessments for clinical trials. A subset of the most reliable measures is recommended for measuring walking and balance in clinical settings.

## Background

Multiple sclerosis (MS) is an autoimmune disease which disrupts the myelin sheath surrounding neurons within the central nervous system [1]. It is estimated that MS affects around 350,000 patients in the United States and more than 2.3 million people worldwide [2, 3]. Symptoms of MS often include mild to severe dysfunction of motor and cognitive faculties such as muscle weakness, spasms, tremors, stiffness, fatigue, deficits in attention and executive functions, and loss of coordination and impaired balance [1]. Persons with MS (PwMS) often report difficulty in walking or standing, with up to 63% of PwMS reporting at least one fall within a 2 to 6 month period [1, 4, 5]. Unfortunately, the diverse symptomology of MS and the lack of quantitative clinical assessments of walking and balance often make it difficult to clinically assess fall risk status of PwMS.

Current clinical assessments for walking and balance difficulties in MS include measures of gait speed based on clinical tests such as the Timed Up and Go or 25 ft walk and relatively subjective measures of balance such as the Berg Balance Test [6]. Unfortunately, many of these scales are limited in their ability to accurately monitor progression of disease or intervention efficacy due to inherent subjectivity, lack of sensitivity in differentiating between groups, and poor reliability [5, 7]. Objective postural measures obtained from motion capture and posturography in PwMS have demonstrated fair to excellent validity and reliability in previous studies [8, 9]. Although effective, motion capture and force platform systems are not practical for use in most clinical settings due to high cost, difficulty of use, and lack of portability.

Wireless inertial sensors are a feasible, low cost alternative tool to assess movement and can be used in any environment [10, 11]. These devices commonly include accelerometers, gyroscopes, magnetometers, or any combination thereof, in order to objectively quantify motor patterns [12]. Such wireless sensors are highly portable with sufficient battery life allowing them to be worn for extended periods of time without constricting movement, which is especially favorable in a clinical or at-home setting [13]. The implementation of such sensors in clinical environments is of particular interest, as these sensors have the potential to enhance objectivity, sensitivity, and reliability of clinical tests [11, 14–16]. Sensor-based measures of postural sway and gait have been found to be sensitive to mobility deficits and reliable in persons with Parkinson’s disease and diabetic neuropathy [17–20]. These findings indicate that wireless inertial sensors can provide a reliable and sensitive measure of walking and balance in clinical settings [11, 19, 20]. Previous work has shown that wireless sensor assessments are sensitive to differences in gait and balance between healthy control subjects and PwMS [21, 22] and are reliable across trials within the same day [23–26]. However, within day reliability testing is not sufficient, as day-to-day fluctuations in performance are common in PwMS [27–29]. While a small subset of gait and balance measures have demonstrated between-day reliability in PwMS [25, 30], to our knowledge there are no previous studies that have determined the between-day test-retest reliability of a comprehensive set of balance and gait measures which includes spatio-temporal and frequency measures taken during an instrumented standing balance and instrumented Timed Up and Go (TUG) tests in PwMS. The lack of reliability testing currently limits the use of this technology for PwMS in clinical and research settings. Additionally, determining the between-day reliability of a comprehensive set of gait and balance measures extracted from wireless sensors will aid in sample size justifications for future studies.

Therefore, the purpose of this study was to determine the between-day test-retest reliability of wireless inertial sensor measures obtained during an instrumented standing balance test and an instrumented TUG test in PwMS. It was hypothesized that the instrumented TUG and instrumented standing balance outcome measures would exhibit strong test-retest reliability in PwMS, as has been previously found in healthy adults, in persons with Parkinson’s disease [20, 31], and in within-day reliability testing [23, 24]. To address the clinical validity of these wireless sensor measures, we also looked at the relationship between the measures and self-report walking and balance function, fall history, and clinical disability. We expected to find significant correlations between the wireless inertial sensor measures and these clinical measures.

## Methods

### Study design

The aim of this study was to determine the between-day test-retest reliability of wireless inertial sensor measures obtained during an instrumented standing balance test and an instrumented Timed Up and Go test in PwMS. The study was performed in a motion analysis laboratory.

### Participants

Fifteen PwMS between 20 and 60 years old and 15 age and gender-matched healthy controls were recruited for this study. All PwMS had relapsing-remitting MS. PwMS were excluded if 1) they were currently prescribed symptom specific medication therapies (i.e. Fampridine) due to its direct effect on gait, 2) if they had experienced a symptom exacerbation in the previous 60 days that required treatment, 3) if they had a Kurtzke Expanded Disability Status Scale (EDSS) [32] greater than 5.5 or were unable to walk a distance of 25 ft without the assistance of a mobility aid. The EDSS assessment for PwMS was completed by a board certified neurologist (author SL) and was completed within 6 months of testing. For both healthy controls and PwMS, participants were excluded if they were women who were pregnant, breastfeeding, or within 3 months post-partum. Subjects were also excluded if they had vestibular impairments, diabetes, or a pre-existing condition that could make exercising difficult (i.e. myocardial infarction, chest pain, unusual shortness of breath, congestive heart failure, etc.). Healthy controls were free of any known neurological or musculoskeletal impairment that would have an adverse effect on their balance or gait. PwMS self-reported how many falls they experienced in the preceding 6 months, with falls being described as “an unexpected event at which the participant comes to rest on the ground, floor, or lower level [33].” Demographic and clinical details for all subjects are shown in Table 1.

**Table 1:** Subject demographics, mean (standard deviation), for healthy controls (HC) and persons with multiple sclerosis (PwMS). All PwMS had relapsing remitting MS

Measure	HC (*n* = 15)	PwMS (*n* = 15)
Gender	12 F/3 M	12 F/3 M
Age (years)	47.8 (9.5)	48.2 (8.7)
BMI (kg/m^2^)	29.01 (6.68)	30.43 (6.98)
EDSS	--	1.89 (0.98)
Years since diagnosis	--	12.2 (5.9)
Self-report # of falls in last 6 months	--	0.3 (0.6)

### Protocol

Subjects were outfitted with 6 wireless inertial sensors (Opal sensors, APDM, Portland, OR, USA) secured by elastic straps during the entirety of testing. The trunk sensor was mounted on the superior trunk over the anterior surface of the sternum, the lumbar sensor was mounted on the inferior trunk over the posterior surface at the L5 level, wrist sensors were mounted bilaterally to the posterior surface of the wrist, and ankle sensors were mounted bilaterally just superior to the ankle joint on the anterior surface of the shank.

During the instrumented standing balance assessment, all participants were instructed to maintain a quiet standing position with arms crossed over their chest and eyes open and looking straight ahead. A constant foot position of 10 cm between the heels was marked for all subjects and maintained all trials. Each trial lasted 30 s and was repeated 3 times. The median value across 3 trials for each instrumented standing balance measure was used for analysis (Table 2).

**Table 2:** Summary of instrumented standing balance outcome measures used in the current study

Measure abbreviation	Description
Jerk	Sway jerk, the time derivative of acceleration (ACC) (m^2^/s^5^)
Distance	Mean distance from center of COP (ACC) trajectory [mm](m/s^2^)
Area	Sway area, computed as area spanned from COP (ACC) per unit of time [mm^2^/s] (m^2^/s^5^)
RMS	Root mean square of COP (ACC) time series [mm](m/s^2^)
Path length	Sway path, total length of COP (ACC) trajectory [mm](m/s^2^)
Range	Range of COP displacement (ACC) [mm](m/s^2^)
Mean velocity	Mean velocity COP = PATH/(trial duration) [m/s]
Mean frequency	Mean frequency = PATH/(2*n*DIST*(trial duration)) (Hz)
95% frequency	95% power frequency, frequency below which 95% of PWR is present (Hz)
Frequency dispersion	Frequency dispersion (Hz)

For the instrumented TUG assessment, subjects were initially seated in a chair with their backs against the seatback. At the start of the test, subjects were given the command “Walk,” which signaled the start of the test. Subjects were instructed to stand up with minimal use of their hands, walk at a normal pace to a point on the floor 7 m in front of them, turn around, walk at a normal pace back to the chair, and sit back down in the chair with minimal use of their hands. The 7-m TUG, sometimes referred to as the extended TUG, allows for a sufficient number of gait cycles necessary for the calculation of the reported gait metrics [20]. Subjects repeated this test 3 times. The median value across 3 trials for each instrumented TUG measure was used for analysis (Table 3).

**Table 3:** Definition of instrumented TUG outcome measures used in the current study

Measure abbreviation	Description
Time	Total time for the subject to complete the TUG (s)
Stride length	Stride length, distance between two consecutive heel contacts, averaged for left and right side. Normalized for height (% of subject height)
Stride velocity	Stride velocity, (% of subject height/s)
Cadence	Stepping rate (Steps/min)
Cycle time	Gait cycle time (GCT), duration of a complete gait cycle (s)
Double support	Double support, percentage of gait cycle with both feet on ground (% of GCT)
Swing time	Average percentage of a gait cycle that either foot is off the ground (% of GCT)
Stance time	Average percentage of gait cycle that either foot is on the ground (% of GCT)
Shank RoM	Shank range of motion, average of left and right (degrees)
Shank velocity	Peak (95%) shank angular velocity, average of left and right (degrees/s)
Arm RoM	Arm swing range of motion, average of the left and right sides (degrees)
Arm velocity	Peak (95%) arm angular velocity, average of left and right (degrees/s)
Trunk hor RoM	Range of motion of the trunk in the horizontal plane (degrees)
Trunk sag RoM	Range of motion of the trunk in the sagittal plane (degrees)
Trunk front RoM	Range of motion of the trunk in the frontal plane (degrees)
Trunk hor velocity	Peak angular velocity of the trunk in the horizontal plane (degrees/s)
Trunk sag velocity	Peak angular velocity of the trunk in the sagittal plane (degrees/s)
Trunk front velocity	Peak angular velocity of the trunk in the frontal plane (degrees/s)

*RoM* range of motion, *hor* horizontal, *sag* sagittal, *front* frontal

All subjects were tested on two separate days with baseline testing performed on day 1 and identical follow-up testing performed on day 2 which was no more than 1 week later. The testing procedures were identical on day 1 and day 2 and no other assessments were done besides the instrumented TUG and instrumented standing balance on either day. Time of day was also kept constant between day 1 and day 2 such that testing began at the exact same time on each day.

Subjects also completed two self-report assessment questionnaires: the 12-item multiple sclerosis walking scale (MSW12) and the activities balance confidence scale (ABC). The MSW12 questionnaire is designed to measure how multiple sclerosis has affected the individual’s walking ability [34]. The ABC questionnaire is designed to measure a person’s confidence that they would not fall while performing a variety of activities [35].

### Data analysis

The wireless sensors used in the current study contain two accelerometers, one gyroscope, and one magnetometer which stream data during the assessments. The wireless sensors used have a preset sample rate of 128 Hz. The two onboard accelerometers have ranges of ±16 g and ±200 g, and resolutions of 14 bits and 17.5 bits respectively. The onboard gyroscope has a range of ±2000 deg/s and a resolution of 16 bits. The onboard magnetometer has a range of ±8 Gauss and a resolution of 12 bits. All measures extracted from the instrumented standing balance and instrumented TUG tests were automatically calculated using Mobility Lab software (APDM, Portland, OR, USA). Thorough explanation and validation of the calculations used for these measures can be found in previous studies [19, 20, 36–38]. The metrics evaluated during the instrumented standing balance and TUG tests have been evaluated previously using a variety of wireless inertial sensor systems in both healthy and pathological populations [15, 17, 19–21, 39].

### Statistical analysis

All statistical analyses were performed using SPSS (Version 20, SPSS Inc., Chicago, IL, USA). Test-retest reliability was assessed using intraclass correlation coefficients (ICC 2,k) [40]. The *p*-value and 95% confidence intervals for each ICC was also determined. ICC values were interpreted as follows: >0.75 was excellent, 0.60–0.74 was good, 0.40–0.59 was fair, <0.40 was poor [41]. Pearson’s correlations examined relationships for: instrumented standing balance measures vs. ABC questionnaire score, instrumented TUG measures vs. MSW12 questionnaire score, instrumented standing balance and instrumented TUG vs. EDSS, and instrumented standing balance and instrumented TUG vs. fall history. Pearson’s correlation coefficients were interpreted as follows: >0.70 was strong, 0.50–0.70 was moderate, 0.30 – 0.50 was weak [42]. An alpha level of 0.05 was used for all statistical tests.

## Results

**Fig. 1:**
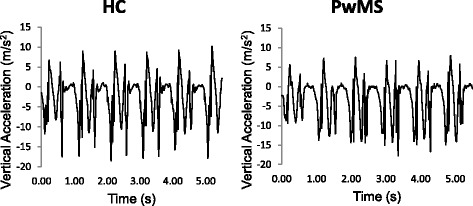
Example time series recorded during standing balance by the lumbar accelerometer for healthy controls (HC) (*left*) and persons with multiple sclerosis (PwMS) (*right*)

MS subjects’ EDSS scores ranged from 1 to 3.5 (Table 1). Descriptive statistics, ICCs and 95% confidence intervals for all instrumented standing balance and instrumented TUG measures are shown in Tables 4 and 5 respectively. All instrumented TUG measures displayed excellent test-retest reliability in PwMS. All but one instrumented TUG measure (stride length ICC = 0.696) displayed excellent (ICC > 0.75) test-retest reliability in HC. Examples of the walking acceleration time series are shown in Fig. 1.

**Table 4:** Descriptive statistics for instrumented standing balance results including ICCs and 95% confidence intervals

	Healthy controls					Persons with multiple sclerosis				
	Day 1 Mean (StD)	Day 2 Mean (StD)	ICC	95% CI bounds		Day 1 Mean (StD)	Day 2 Mean (StD)	ICC	95% CI bounds	
			*ρ*	Lower	Upper			*ρ*	Lower	Upper
Jerk	0.096 (0.042)	0.084 (0.045)	**0.880**	0.642	0.960	0.105 (0.065)	0.126 (0.110)	**0.858**	0.578	0.952
Distance	0.058 (0.021)	0.055 (0.014)	**0.788**	0.368	0.929	0.067 (0.022)	0.070 (0.034)	**0.919**	0.759	0.973
Area	0.003 (0.001)	0.003 (0.001)	**0.832**	0.500	0.944	0.004 (0.002)	0.004 (0.004)	**0.891**	0.675	0.963
RMS	0.071 (0.025)	0.065 (0.017)	**0.790**	0.375	0.929	0.078 (0.024)	0.083 (0.039)	**0.895**	0.687	0.965
Path length	4.893 (0.953)	4.769 (1.255)	**0.895**	0.687	0.965	5.516 (1.631)	5.543 (1.936)	**0.884**	0.655	0.961
Range	0.369 (0.101)	0.345 (0.104)	**0.731**	0.200	0.910	0.384 (0.103)	0.407 (0.192)	**0.809**	0.430	0.936
Mean velocity	0.140 (0.063)	0.143 (0.056)	**0.699**	0.105	0.899	0.152 (0.085)	0.164 (0.075)	**0.807**	0.426	0.935
Mean frequency	0.497 (0.150)	0.466 (0.108)	**0.795**	0.389	0.931	0.461 (0.130)	0.440 (0.121)	**0.900**	0.703	0.967
95% frequency	1.735 (0.474)	1.718 (0.302)	**0.672**	0.024	0.890	1.630 (0.316)	1.538 (0.375)	**0.853**	0.561	0.951
Frequency dispersion	0.783 (0.036)	0.781 (0.037)	0.151	−1.528	0.715	0.748 (0.040)	0.753 (0.039)	0.438	−0.675	0.811

ICC values in **boldface** show at least “good” reliability (ICC > 0.60)

**Table 5:** Descriptive statistics for instrumented TUG results including, ICCs, and 95% confidence intervals ICC values in **boldface** show at least “good” reliability (ICC > 0.60)

	Healthy controls					Persons with multiple sclerosis				
	Day 1 Mean (StD)	Day 2 Mean (StD)	ICC	95% CI bounds		Day 1 Mean (StD)	Day 2 Mean (StD)	ICC	95% CI bounds	
			*ρ*	Lower	Upper			*ρ*	Lower	Upper
Time	17.93 (2.31)	17.58 (2.19)	**0.939**	0.819	0.980	17.05 (2.52)	16.35 (2.30)	**0.965**	0.894	0.988
Stride length	85.67 (5.46)	85.20 (7.34)	**0.696**	0.096	0.898	84.39 (3.63)	85.35 (3.82)	**0.908**	0.726	0.969
Stride velocity	81.98 (6.89)	81.69 (8.36)	**0.863**	0.592	0.954	83.71 (8.13)	85.76 (9.23)	**0.948**	0.846	0.989
Cadence	114.95 (6.69)	115.13 (6.42)	**0.959**	0.878	0.986	118.60 (8.53)	120.47 (10.34)	**0.968**	0.905	0.989
Cycle time	1.05 (0.07)	1.05 (0.06)	**0.958**	0.874	0.986	1.02 (0.07)	1.00 (0.09)	**0.962**	0.886	0.987
Double support	23.13 (3.91)	24.09 (4.62)	**0.819**	0.461	0.939	21.93 (4.63)	22.46 (4.50)	**0.864**	0.595	0.954
Swing time	38.43 (1.95)	37.96 (2.31)	**0.818**	0.457	0.939	39.03 (2.30)	38.77 (2.25)	**0.864**	0.595	0.954
Stance time	61.57 (1.95)	62.04 (2.31)	**0.818**	0.457	0.939	60.97 (2.30)	61.23 (2.25)	**0.864**	0.595	0.954
Shank RoM	81.15 (5.73)	79.96 (7.76)	**0.767**	0.305	0.922	80.13 (5.17)	80.21 (4.52)	**0.963**	0.890	0.988
Shank velocity	405.13 (33.93)	401.07 (47.67)	**0.809**	0.430	0.936	416.07 (40.80)	420.80 (43.08)	**0.923**	0.771	0.974
Arm RoM	26.29 (9.13)	25.63 (11.24)	**0.874**	0.624	0.958	23.33 (9.67)	28.31 (13.13)	**0.888**	0.665	0.962
Arm velocity	208.73 (57.25)	212.20 (55.69)	**0.848**	0.548	0.949	233.47 (58.33)	242.40 (61.41)	**0.947**	0.842	0.982
Trunk hor RoM	4.90 (2.05)	4.91 (2.00)	**0.885**	0.656	0.961	5.43 (1.78)	5.33 (1.77)	**0.925**	0.776	0.975
Trunk sag RoM	5.03 (1.56)	4.57 (1.56)	**0.879**	0.639	0.959	4.72 (2.19)	4.32 (1.25)	**0.777**	0.336	0.925
Trunk front RoM	8.07 (2.40)	8.07 (2.29)	**0.938**	0.816	0.979	9.84 (2.90)	9.49 (2.99)	**0.975**	0.925	0.992
Trunk hor velocity	25.33 (9.24)	25.73 (7.45)	**0.849**	0.551	0.949	29.11 (8.85)	29.39 (9.46)	**0.909**	0.729	0.969
Trunk sag velocity	42.36 (18.94)	36.75 (17.07)	**0.968**	0.904	0.989	39.35 (26.89)	37.63 (17.50)	**0.902**	0.708	0.967
Trunk front velocity	40.25 (11.08)	41.01 (11.36)	**0.855**	0.569	0.951	45.53 (15.77)	44.07 (13.77)	**0.957**	0.872	0.986

**Fig. 2:**
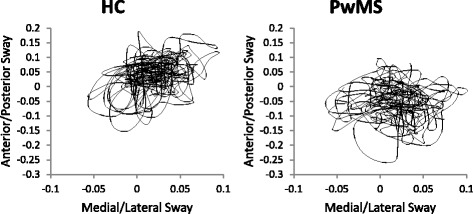
Example time series of walking portion of instrumented 7 m Timed Up and Go test recorded by right foot accelerometer for healthy controls (HC) (*left*) and persons with multiple sclerosis (PwMS) (*right*)

For instrumented standing balance measures, there was a larger range of ICC values for both PwMS and HC. In PwMS, all instrumented standing balance measures, except one (frequency dispersion ICC = 0.438), displayed excellent test-retest reliability. In HC, all measures displayed excellent test-retest reliability except three measures that displayed good test-retest reliability (range ICC = 0.731, mean velocity ICC = 0.699, 95% frequency ICC = 0.672) and one measure displayed poor test-retest reliability (frequency dispersion ICC = 0.151). Examples of the standing balance acceleration time series are shown in Fig. 2.

There were no significant correlations between instrumented standing balance outcome measures and the ABC questionnaire scores (Table 6), or between the instrumented TUG outcome measures and the MSW12 questionnaire scores (Table 7). EDSS scores were moderately correlated with four instrumented standing balance variables; distance (*r* = −0.533), RMS (*r* = −0.549), range (*r* = −0.543), and mean frequency (*r* = 0.538) (Table 6). EDSS scores were moderately correlated with four instrumented TUG variables; stride velocity (*r* = 0.609), cadence (*r* = 0.674), cycle time (*r* = −0.655), and shank velocity (*r* = 0.624) (Table 7). There were no significant correlations between instrumented standing balance outcome measures and self-reported number of falls (Table 6). Self-reported number of falls was moderately correlated with stride velocity (*r* = −0.557), cadence (*r* = −0.641) and cycle time (*r* = 0.652) (Table 7).

**Table 6:** Correlations between instrumented standing balance measures and ABC questionnaire scores in persons with multiple sclerosis only

	Measure vs. ABC		Measure vs. EDSS		Measure vs. Falls	
Instrumented standing balance measure	*r*	*p*	*r*	*p*	*r*	*p*
Jerk	0.402	0.155	−0.234	0.421	0.254	0.381
Distance	0.457	0.100	−0.533*	0.050	0.198	0.498
Area	0.328	0.253	−0.376	0.185	0.222	0.446
RMS	0.477	0.085	−0.549*	0.042	0.220	0.450
Path length	0.346	0.225	−0.206	0.479	0.195	0.504
Range	0.498	0.070	−0.543*	0.045	0.250	0.389
Mean velocity	0.279	0.335	−0.435	0.120	0.075	0.798
Mean frequency	−0.269	0.352	0.538*	0.047	−0.080	0.785
95% frequency	−0.234	0.420	0.197	0.501	−0.133	0.650
Frequency dispersion	0.255	0.379	−0.525	0.054	0.134	0.647

*EDSS* expanded disability status scale

*Significant correlation, *p* < 0.05

**Table 7:** Correlations between instrumented TUG measures and MSW12 questionnaire scores in persons with multiple sclerosis only

	Instrumented TUG vs. MSW12		Instrumented TUG vs. EDSS		Instrumented TUG vs. Falls	
Instrumented TUG Measure	*r*	*p*	*r*	*p*	*r*	*p*
Time	−0.200	0.492	−0.290	0.314	0.279	0.335
Stride length	0.212	0.468	0.265	0.360	−0.198	0.498
Stride velocity	0.320	0.265	0.609*	0.021	−0.557*	0.039
Cadence	0.309	0.282	0.674*	0.008	−0.641*	0.014
Cycle time	−0.330	0.249	−0.655*	0.011	0.652*	0.011
Double support	−0.143	0.625	0.061	0.835	−0.133	0.650
Swing time	0.147	0.617	−0.054	0.854	0.136	0.643
Stance time	−0.147	0.617	0.054	0.854	−0.136	0.643
Shank RoM	0.046	0.877	0.225	0.438	−0.221	0.448
Shank velocity	0.253	0.384	0.624*	0.017	−0.449	0.107
Arm RoM	−0.212	0.466	−0.256	0.377	0.032	0.914
Arm velocity	−0.057	0.847	−0.174	0.553	−0.431	0.124
Trunk hor RoM	−0.455	0.102	−0.007	0.980	0.028	0.923
Trunk sag RoM	−0.089	0.761	0.013	0.966	−0.378	0.183
Trunk front RoM	−0.516	0.059	−0.489	0.076	0.311	0.279
Trunk hor velocity	−0.417	0.138	0.000	1.000	−0.151	0.607
Trunk sag velocity	0.044	0.882	0.073	0.805	−0.339	0.236
Trunk front velocity	−0.331	0.248	−0.116	0.314	0.060	0.839

*EDSS* expanded disability status scale

*Significant correlation, *p* < 0.05

## Discussion

The current study determined the between-day test-retest reliability of a comprehensive set of wireless sensor measures from instrumented standing balance test and an instrumented Timed-Up and Go test on PwMS. Almost all of the instrumented standing balance and instrumented TUG measures exhibited good to excellent reliability across the two separate testing days. Previous work has shown that wireless sensor based assessments are sensitive to gait and balance deficits in healthy adults [43], patients with Parkinson’s disease [19, 20], and PwMS [21, 22]. Additionally, many of the measures obtained from these wireless sensors exhibit good to excellent test-retest reliability in aging adults [26] and patients with Parkinson’s disease [19, 20]. To date, within-day reliability studies using wireless sensor measures have been performed in PwMS [23, 24], but between-day testing has only been performed in a small subset of wireless sensor measures [25, 30]. The current analysis builds upon previous work by determining the between-day reliability of a comprehensive set of gait and balance measures in persons with multiple sclerosis.

Our results provide support for using wireless inertial sensors to reliably measure gait and balance in persons with multiple sclerosis. Our results show that the test-retest reliability for instrumented standing balance outcome measures was best for spatio-temporal measures such as path length and jerk, while the frequency measures such as frequency dispersion were less reliable. The lowered reliability in the frequency measures during the standing balance assessment has been observed in previous work [19, 44] and may be due to variations in subjects’ balance strategies between the testing sessions. Subjects’ foot positioning was normalized between testing sessions, however this does not fully control for balance strategy differences such as swaying about the ankle 1 day, or using the hip more on another day. While these different strategies may induce changes in frequency content of sway, both allow the subjects to achieve sufficient balance performance. Almost all instrumented TUG measures exhibited excellent test-retest reliability, with the only exception being stride length in HC, which showed good test-retest reliability.

The ICCs for the HC subjects were slightly lower than those for PwMS. Previous work has shown similar trends, with HC subjects having lower ICCs compared to patients with Parkinson’s disease [19]. There is, however, substantial overlap of the 95% confidence intervals between the two groups for every ICC value indicating that the ICC differences are likely not significant. Nevertheless, this trend is likely due to a higher amount of intra-subject variability in our MS subjects’ walking and balance performance without an increase in performance variability between the two testing sessions. Our descriptive statistics also reflect increased variability as the standard deviations for the instrumented standing balance and instrumented TUG measures tended to be larger in PwMS. Previous work has noted that PwMS have altered variability during gait potentially due to deficits such as gait ataxia, which causes problems in the control of gait and results in an increase in random variability during gait [10, 22]. Previous work examining clinical balance assessments, questionnaires, and a subset of wireless sensor assessments have also shown good to excellent test-retest reliability in PwMS [30, 45], which are in agreement with the current findings.

We expected to find correlations between some of the wireless inertial sensor measures and the questionnaires, fall history, and clinical disability. However there were no significant correlations found between any of the instrumented standing balance measures and. the ABC questionnaire or between the instrumented TUG measures and MSW12 questionnaire. The ABC questionnaire is designed to assess a person’s balance confidence in everyday life, while the MSW12 questionnaire is designed to measure how MS has affected the individual’s walking [34, 35]. Lack of correlation between wireless inertial sensor measures and self-report questionnaires could be due to the subjective questions and lack of sensitivity of the questionnaires [46]. Since the PwMS who participated in the current study were classified with mild impairment from their EDSS score, similar to previous studies [21, 47], it is possible the questionnaires were simply not sensitive enough to distinguish small inter-subject differences. Specifically, even the most impaired subject in our sample may have had very similar self-perceived balance and mobility scores compared to the least impaired subject in our sample. However, there were significant correlations between both instrumented standing balance measures (distance, RMS, range, and mean frequency) and instrumented TUG measures (stride velocity, cadence, cycle time, and shank velocity) and EDSS which indicates good clinical validity between some wireless inertial sensor measures and the gold standard clinical disability scale. For example, higher cadence measured from the instrumented TUG assessment in PwMS was correlated with a higher EDSS as assessed by a neurologist, indicating a relationship between these measures. Three instrumented TUG measures (stride velocity, cadence, and gait cycle time) also showed significant correlations with fall history. Because previous history of falls is a primary predictor of future falls [7], it is possible that stride velocity, cadence, gait cycle time measured during the instrumented TUG could be monitored on a regular basis and used to identify changes in individuals’ functional status or risk of future falls. Longitudinal studies evaluating these outcomes in PwMS are needed to confirm the use of wireless inertial sensor measures as fall predictors.

The current study has a relatively small sample size and the PwMS were high functioning with low disability, which limits the ability to generalize the findings of the current study to all individuals with MS. However, similar previous studies have used similar sample sizes [19, 25, 26], and even within this small sample, 26 out of 27 metrics taken from the instrumented standing balance and instrumented TUG assessments exhibited good to excellent reliability (ICC range 0.693–0.962).

## Conclusions

The current study provides important information concerning the test-retest reliability of measures extracted from an instrumented TUG and instrumented standing balance in PwMS. The test-retest reliability results from the current study can be used in future studies when power estimations are needed to determine a required sample size. A majority of the outcome measures from the instrumented TUG and instrumented standing balance exhibited good to excellent reliability. For PwMS, the mean distance from the center of pressure (distance) was the most reliable outcome measure from the instrumented standing balance assessment, while range of motion of the trunk in the frontal plane (trunk front RoM) was the most reliable outcome measure from the instrumented TUG. Overall these assessments provide reliable measures of walking and postural control which can be used as screening protocols or mobility assessment outcome measures.

## Abbreviations

ABC: Activities balance confidence scale
BMI: Body mass index
EDSS: Kurtzke Expanded Disability Status Scale
HC: Healthy control subjects
ICC: Intraclass correlation coefficients
MS: Multiple sclerosis
MSW12: 12-item multiple sclerosis walking scale
PwMS: Persons with multiple sclerosis
Trunk front RoM: Range of motion of the trunk in the frontal plane
TUG: Timed Up and Go
